# Clinical Significance and Potential Mechanisms of ATP Binding Cassette Subfamily C Genes in Hepatocellular Carcinoma

**DOI:** 10.3389/fgene.2022.805961

**Published:** 2022-03-07

**Authors:** Xin Zhou, Jia-mi Huang, Tian-man Li, Jun-qi Liu, Zhong-liu Wei, Chen-lu Lan, Guang-zhi Zhu, Xi-wen Liao, Xin-ping Ye, Tao Peng

**Affiliations:** ^1^ Department of Hepatobiliary Surgery, The First Affiliated Hospital of Guangxi Medical University, Nanning, China; ^2^ Key Laboratory of Early Prevention and Treatment for Regional High Frequency Tumor (Guangxi Medical University), Ministry of Education, Nanning, China; ^3^ Department of Hepatobiliary Surgery, The Sixth Affiliated Hospital of Guangxi Medical University, Yulin, China

**Keywords:** HCC (hepatic cellular carcinoma), ABCC gene family, prognosis (carcinoma), nomogram, GSEA (gene set enrichment analysis)

## Abstract

The purpose of this investigation was to assess the diagnostic and prognostic significance of *ATP binding cassette subfamily C (ABCC)* genes in hepatocellular carcinoma (HCC). The Student t-test was used to compare the expression level of *ABCCs* between HCC and paraneoplastic tissues. Receiver operating characteristic curve (ROC) analysis was applied for diagnostic efficiency assessment. The Kaplan–Meier method and Cox proportional hazards model were respectively applied for survival analysis. Genes with prognostic significance were subsequently used to construct prognostic models. From the perspective of genome-wide enrichment analysis, the mechanisms of prognosis-related ABCC genes were attempted to be elaborated by gene set enrichment analysis (GSEA). It was observed in the TCGA database that *ABCC1*, *ABCC4*, *ABCC5*, and *ABCC10* were significantly upregulated in tumor tissues, while *ABCC6* and *ABCC7* were downregulated in HCC tissues. Receiver operating characteristic analysis revealed that *ABCC7* might be a potential diagnostic biomarker in HCC. *ABCC1*, *ABCC4*, *ABCC5*, and *ABCC6* were significantly related to the prognosis of HCC in the TCGA database. The prognostic significance of *ABCC1*, *ABCC4*, *ABCC5*, and *ABCC6* was also observed in the Guangxi cohort. In the Guangxi cohort, both polymerase chain reaction and IHC (immunohistochemical) assays demonstrated higher expression of ABCC1, ABCC4, and ABCC5 in HCC compared to liver tissues, while the opposite was true for ABCC6. GSEA analysis indicated that *ABCC1* was associated with tumor differentiation, nod-like receptor signal pathway, and so forth. It also revealed that *ABCC4* might play a role in HCC by regulating epithelial-mesenchymal transition, cytidine analog pathway, met pathway, and so forth. *ABCC5* might be associated with the fatty acid metabolism and KRT19 in HCC. *ABCC6* might impact the cell cycle in HCC by regulating E2F1 and myc. The relationship between ABCC genes and immune infiltration was explored, and ABCC1,4,5 were found to be positively associated with infiltration of multiple immune cells, while ABCC6 was found to be the opposite. In conclusion, *ABCC1*, *ABCC4*, *ABCC5*, and *ABCC6* might be prognostic biomarkers in HCC. The prognostic models constructed with *ABCC1*, *ABCC4*, *ABCC5*, and *ABCC6* had satisfactory efficacy.

## Background

Hepatocellular carcinoma (HCC) generally followed cirrhosis given rise by metabolic disorder (Yang et al., 2019), chronic ethanol intake (Llovet et al., 2016), and hepatitis virus infection (Fujiwara et al., 2018). The leading metabolic risk factor for HCC is non-alcoholic fatty liver disease (NAFLD) (Zhang, 2018), which is mainly related to obesity and type 2 diabetes. Currently, it is acknowledged that hepatitis B virus (HBV) and hepatitis C virus (HCV) were the main infectious etiologies for cirrhosis and HCC. It is worth mentioning that viral hepatitis could skip cirrhosis and induced HCC directly and independently (El-Serag, 2012; Levrero and Zucman-Rossi, 2016). Besides the aforementioned factors, the intake of Aflatoxin B1 (AFB1) was also demonstrated to be related to HCC (Rushing and Selim, 2019). People in specific regions entailing relatively high exposure to Aflatoxin B1 were accompanied by high incidence and mortality of HCC (Long et al., 2008; Wogan et al., 2012; Zhang W et al., 2017). More than 8 million new cases of liver cancer occurred worldwide each year, which directly or indirectly gave rise to more than 4 million deaths worldwide each year (Torre et al., 2015; Bray et al., 2018). Asia has the highest incidence of liver cancer in the world, particularly in China, which accounts for almost half the global cases (Akinyemiju et al., 2017). At the same time, Asia is the high-incidence area of HBV and HCV (Gower et al., 2014; Polaris Observatory Collaborators, 2018). The main treatment methods of liver cancer mainly include surgical resection, transcatheter arterial chemoembolization (TACE), ablation, liver transplantation, radiotherapy, and so forth (Fattovich et al., 2004). Sorafenib, the multi-kinase inhibitor, is one of first-line drugs approved for the treatment of advanced HCC. Although it can improve survival, the long-term survival of HCC patients is limited due to the drug resistance. Hence, the discovery of new hub genes for developing HCC-targeted drugs and specific genes that improve and maintain drug susceptibility might be hopeful for advanced-stage HCC patients.

The ATP binding cassette subfamily C (ABCC) subfamily includes 13 members whose protein products take effect in transporters with different functional profiles, including ion transport, cell surface receptor, and toxin secretion activity (Childs and Ling, 1994; Dean and Allikmets, 2001; Robey et al., 2018; Yamada et al., 2018). The ATP-binding domain of the ABCC product possesses distinctive conserved motifs (Walker A and B motifs), which are separated by an uncertain sequence of around 100 amino acids (Dean et al., 2001). The distinctive interval and conserved motifs distinguish ABCC members from other ATP-binding proteins (Higgins et al., 1986). Genetic variations in these genes are substantiated in numerous research studies to be the cause or contributor to a variety of complex human diseases, including cystic fibrosis, neurological diseases, defects in cholesterol and bile transport, and drug responses. The ABCC subfamily plays an important role in the pharmacokinetics of endogenous and exogenous compounds. Studies have shown that the members of the ABCC family could transport drugs to the extracellular substances by virtue of ATP energy (Chen and Tiwari, 2011; Keppler, 2011; Leslie, 2012).

## Methods

### Data Acquisition and Specimen Collection

RNA-Seq data (FPKM) of 412 samples, 362 tumors, and 50 paraneoplastic tissues were acquired from the TCGA database (https://portal.gdc.cancer.gov/, accessed on 22 December 2019). The *limma* package was employed for normalization of this RNA-Seq data in R. Matched prognostic/clinicopathologic data of these 362 patients were acquired from UCSC Xena (http://xena.ucsc.edu/, accessed on 23 December 2019).

The HCC tissues and matched paracancer tissues of 102 patients hospitalized in the first affiliated hospital of Guangxi Medical University from September 2016 to December 2018 were collected after informed consent was obtained. Among them, excised tissues during surgery of 72 patients were well preserved in the Department of Pathology. Tissue slices of these patients were obtained from the Department of Pathology.

### Expression Difference and Diagnostic Efficiency Analysis of ABCC Genes

The expression levels of *ABCCs* in HCC and paraneoplastic tissues were extracted from the RNA-Seq Chip matrix in the TCGA database. The normality test was assessed using the Kolmogorov–Smirnov normality test. Student’s t-test was used to assess the statistical significance of *ABCC* genes’ expression between HCC and paraneoplastic tissues. The area under the curve (AUC) of the receiver operating characteristic curve (ROC) was used to access the diagnostic efficiency of each *ABCC* gene in HCC. AUC > 0.8 with *p* < 0.05 was considered as satisfactory diagnostic performance (Hosmer et al., 2013).

### Immunohistochemistry

Tissue sections were sequentially placed in xylene and graded concentrations of ethanol to achieve hydration. Antigens were repaired with a pH 6.0 citrate repair solution (ZSGB-BIO, Beijing, China). Subsequent antigen–antibody reactions and color development reactions were performed with the help of a universal two-step detection kit (Mouse/Rabbit Enhanced Polymer Detection System). Immunohistochemical scores were assessed by two experienced pathologists. Antibodies for ABCC1, ABCC4, ABCC5, and ABCC6 were diluted according to the recommended concentrations of the manufacturer (Proteintech, Wuhan, China).

### Prognostic Significance Assessment of ABCC Genes

The patients in the TCGA database were divided into two groups in terms of the median value of each *ABCC* gene expression for survival analysis. The Kaplan–Meier method with a log-rank test was applied to assess the prognostic significance of each *ABCC* gene. The Cox proportional hazards model was applied to adjust the bias caused by prognosis-related clinicopathologic factors.

In terms of survival analysis results in the TCGA database, the prognostic significance of *ABCC1*, *ABCC4*, *ABCC5*, and *ABCC6* was further validated in the Guangxi cohort.

For better predicting the prognosis and evaluating the combined effect of *ABCCs*, prognosis-related *ABCCs* (*ABCC1*, *ABCC4*, *ABCC5*, and *ABCC6*) were integrated in pairs into combined effect survival analysis. The patients were divided into four groups in terms of the expression level of *ABCCs* with details displayed in Table 2. The Kaplan–Meier method with the log-rank test and Cox proportional hazards model were applied to assess the prognostic significance.

### Nomogram

Independent prognostic factors, including *ABCCs* and clinicopathologic features, were integrated to construct the nomogram in R with the *rms* package (Iasonos et al., 2008). In the nomogram, the risk degree of each variable in the nomogram was displayed by the integration line, and the total risk score is obtained by adding up the risk value of each variable (Zhang Z et al., 2017). The model was validated for calibration and discrimination using the *bootstrap* method (Wang et al., 2013).

### Prognostic Signature Construction

The Cox proportional hazards model was used to assess the risk coefficient of *ABCCs* in overall survival. Then, the prognostic signature was constructed in terms of the expression of *ABCCs* and the corresponding risk coefficient. The formula of prognostic signature construction is as follows: $\text{Risk}\;\text{score}=\overset{N}{\underset{1}{\sum }}\left(ExpVluei*\beta i\right)$ (Chen M et al., 2017). N is the number of prognostic genes. ExpVluei is the expression value of each *ABCC* gene. βi is the risk coefficient of the corresponding *ABCC* gene. A time-dependent ROC curve was constructed in R (version 3.6.2; www.r-project.org) with the *survivalROC* package to evaluate the availability of this prognostic signature (Chen M et al., 2017).

### Biological Functional Exploration of ABCC Genes

The Gene Ontology (GO) database, the integrated database of calculable information about the functions of genes, was comprehensively used for identifying unique biological properties of high-throughput transcriptome or genome data (The Gene Ontology Consortium, 2017; Chen L et al., 2017). KEGG is a collection of databases dealing with genomes, diseases, biological pathways, drugs, and chemical materials (Kanehisa et al., 2017). DAVID (The Database for Annotation, Visualization, and Integrated Discovery, https://david.ncifcrf.gov/) is an online bioinformatics tool to access the GO database and the KEGG database (Long et al., 2008). DAVID was used to access the enrichment of biological functions and pathways of ABCC genes in this investigation. Then, the enrichment biological functions and pathways were visualized in R Studio (Version 1.2.5033) with packages *Goplot*, *Hmisc*, and *ggplot2* (Nolan et al., 2013; Ito and Murphy, 2013). The Biological Networks Gene Ontology tool (BiNGO) is an open-source online database, which was employed to determine the significantly overrepresented GO terms of ABCC genes (Maere et al., 2005). Functions and interactions of ABCC genes were performed in Genemania (http://genemania.org/. accessed on 11 August 2020) and STRING (https://string-db.org/. accessed on 11 August 2020), respectively (Szklarczyk et al., 2015; Luo et al., 2020).

### Gene Set Enrichment Analysis (GSEA)

GSEA is software with additional resources for analyzing, annotating, and interpreting standardized chip matrices. In this investigation, GSEA enrichment was used to analyze the enriched biological pathways of *ABCC1*, *ABCC4*, *ABCC5*, and *ABCC6* in the TCGA database. The Oncogenic Signatures c2.all.v7.1.symbols.gmt data set was adopted as the reference data set. The biological pathways exported from GSEA with *p* < 0.05 and FDR < 0.25 were considered as significant results.

### Correlation Analysis of Tumor-Infiltrating Immune Cells and ABCC Gene Expression

TIMER (http://timer.cistrome.org/) is a comprehensive resource for the systematical analysis of immune infiltrates across diverse cancer types, which provides immune infiltrates abundances estimated by multiple immune deconvolution methods. In this investigation, TIMER was accessed to explore the correlation between infiltrating immune cells and *ABCC* expression in HCC.

## Results

### Expression and Diagnostic Efficiency of ABCC Genes in HCC

**FIGURE 1:**
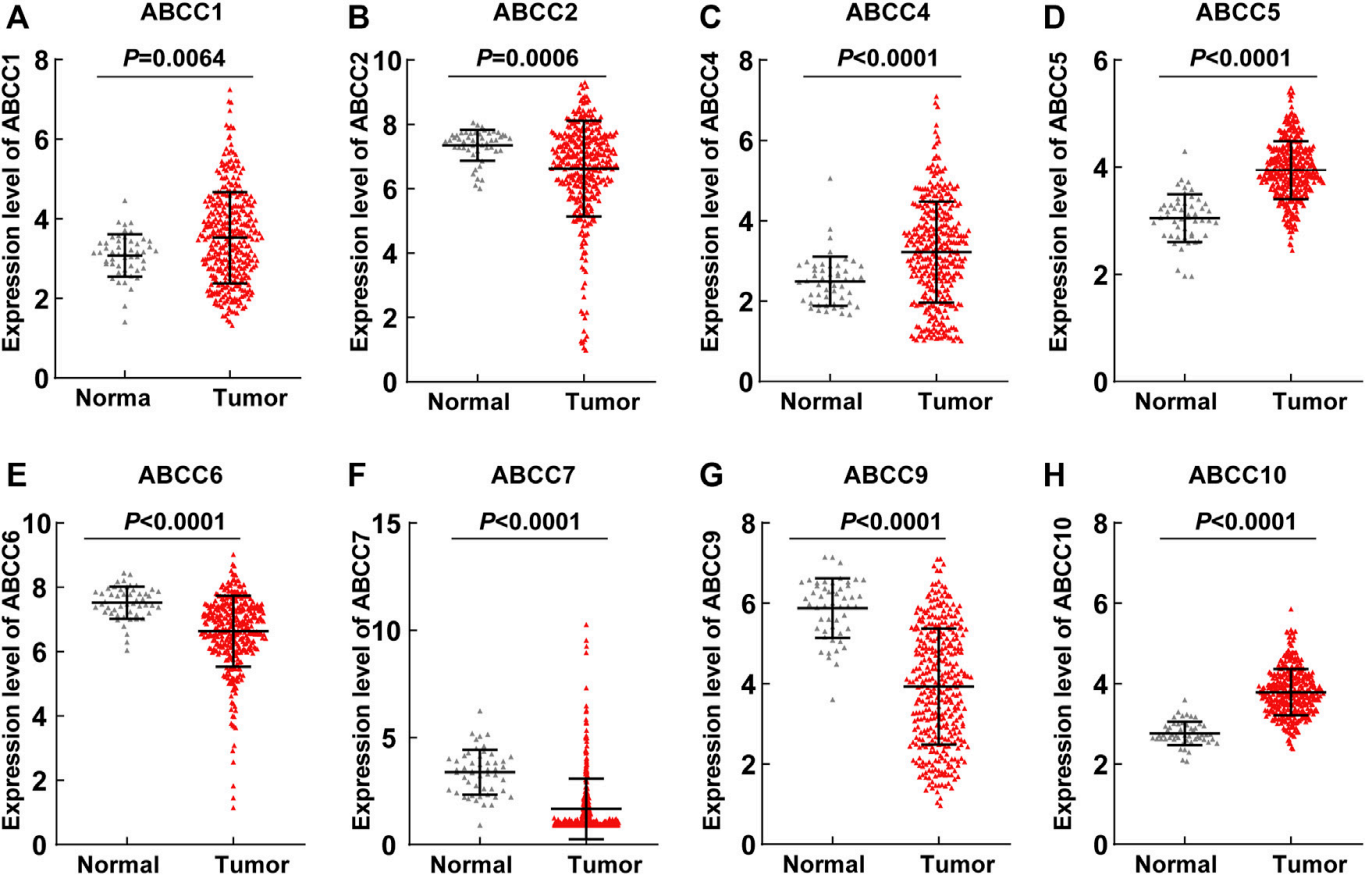
*ABCC1*, *ABCC2*, *ABCC4*, *ABCC5*, *ABCC6*, *ABCC7*, *ABCC9*, and *ABCC10* were differently expressed between the HCC tissues and paraneoplastic tissues based on the RNA-seq data of the TCGA database: **(A)** *ABCC1*, **(B)** *ABCC2*, **(C)** *ABCC4*, **(D)** *ABCC5*, **(E)** *ABCC6*, **(F)** *ABCC7*, **(G)** *ABCC9*, and **(H)** *ABCC10*.

**FIGURE 2:**
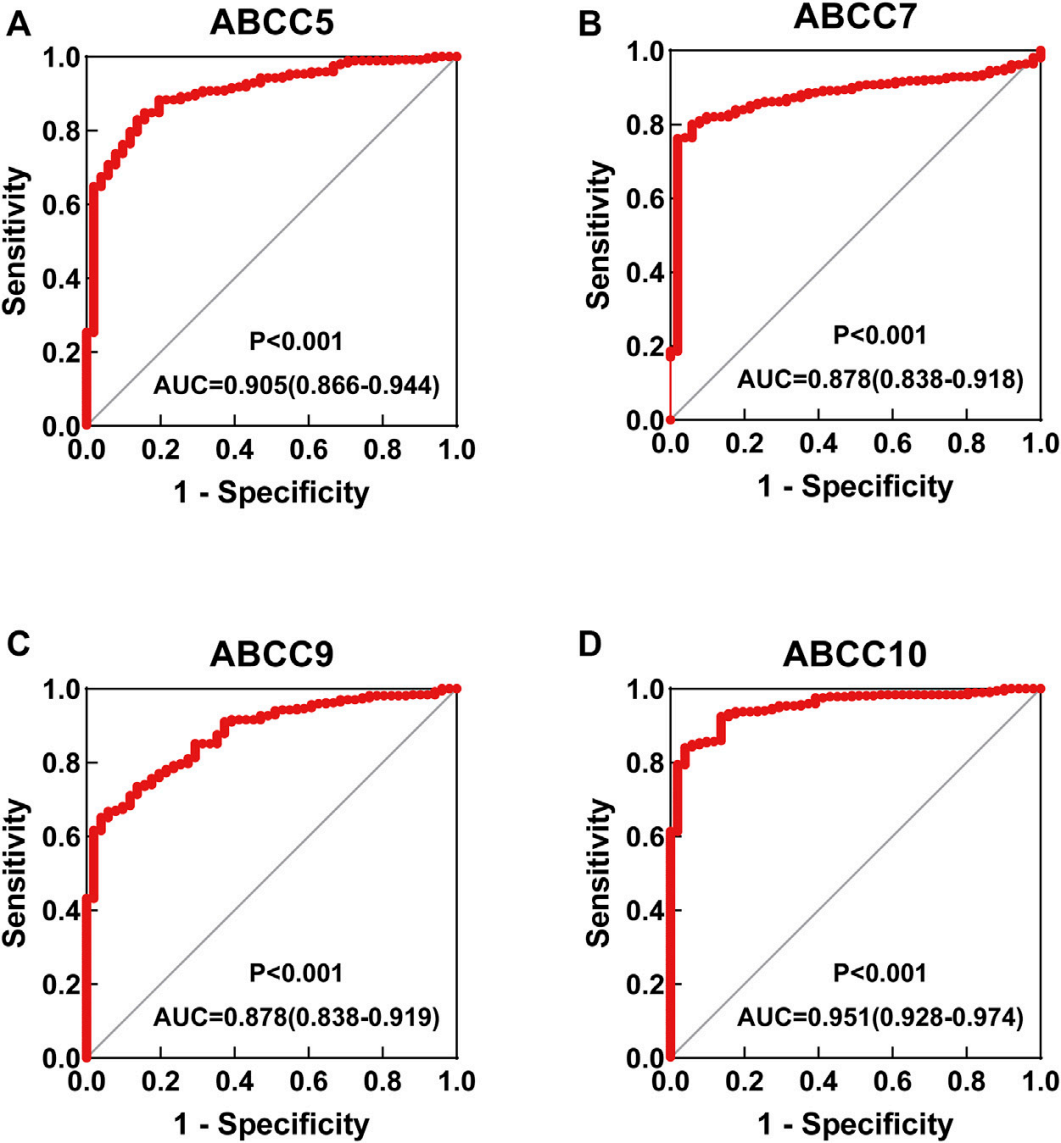
ROC curve for ABCC genes in the TCGA database: **(A)** *ABCC5*, **(B)** *ABCC7*, **(C)** *ABCC9*, and **(D)** *ABCC10*.

Several *ABCC* genes were discovered to be differentially expressed in HCC and paraneoplastic tissues based on the RNA-seq data of the TCGA database. *ABCC1*, *ABCC4*, *ABCC5*, and *ABCC10* (Figures 1A,C,D,H) were significantly higher expressed in HCC tissues, but *ABCC2*, *ABCC6*, *ABCC7*, and *ABCC9* (Figures 1B,E–G) were significantly lower expressed in HCC tissues. No significant differences in the expression of ABCC3, ABCC8, ABCC11, ABCC12, and ABCC13 were observed between liver and HCC tissues (Supplementary Figures S1A–E). The diagnostic efficacy of the genes differentially expressed between HCC and paraneoplastic tissues was subsequently evaluated using ROC curve analysis. Among them (*ABCC1*, *ABCC2*, *ABCC4*, *ABCC5*, *ABCC6*, *ABCC7*, *ABCC9*, and *ABCC10*), high diagnostic efficiencies of *ABCC5* (AUC = 0.905, *p* < 0.001), *ABCC7* (AUC = 0.878, *p* < 0.001), *ABCC9* (AUC = 0.878, *p* < 0.001), and *ABCC10* (AUC = 0.951, *p* < 0.001) (Figures 2A–D) were observed in HCC. The results of the diagnostic efficacy analysis of other ABCC genes are shown in Supplementary Figures S2A–I.

### Prognostic Significance of ABCC Genes

**FIGURE 3:**
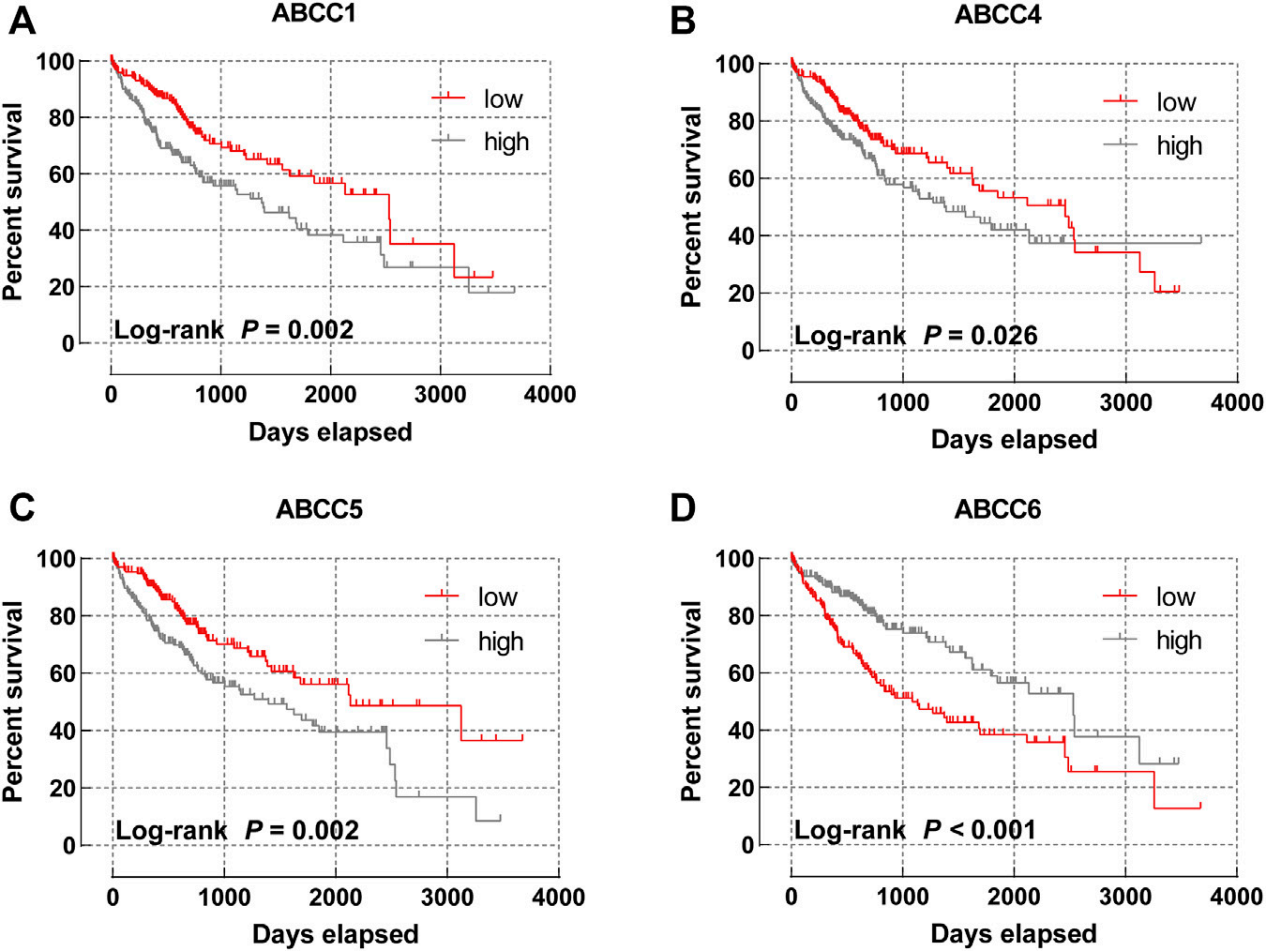
Survival curve of ABCC genes in the TCGA database: **(A)** *ABCC1*, **(B)** *ABCC4*, **(C)** *ABCC5*, and **(D)** *ABCC6.*

Subsequently, the prognostic significance of *ABCC* genes was systematically discussed. The clinicopathologic characteristics of 362 HCC tissues in the TCGA database are displayed in Supplementary Table S1. The expression levels of *ABCC1* (log-rank *p* = 0.002, adjusted *p* = 0.008, adjusted HR = 1.656), *ABCC4* (log-rank *p* = 0.026, adjusted *p* = 0.038, adjusted HR = 1.479), *ABCC5* (log-rank *p* = 0.002, adjusted *p* = 0.001, adjusted HR = 1.928), and *ABCC6* (log-rank *p* < 0.001, adjusted *p* = 0.001, adjusted HR = 0.534) were significantly associated with the overall survival of HCC patients in univariate and multivariate survival analysis (Table 1; Figures 3A–D). In terms of the prognostic value of a single ABCC gene, patients with high expression of *ABCC1*, *ABCC4*, or *ABCC5* tend to be with a shorter median survival time, while high-expression *ABCC6* was associated with longer survival. The results of survival analysis of other ABCC genes are shown in Supplementary Figures S3A–I.

**TABLE 1:** Survival analysis results of ABCC genes in the TCGA database.

Gene expression	Patients (*n* = 362)	Overall survival				
		Number of events	Crude HR (95% CI)	Crude P	Adjusted HR (95% CI)	Adjusted P §
ABCC1						
Low	181	51	1		1	
High	181	78	1.759 (0.235–2.504)	0.002	1.656 (1.137–2.410)	0.008
ABCC2						
Low	181	64	1		1	
High	181	65	1.079 (0.761–1.529)	0.670	1.210 (0.833–1.758)	0.317
ABCC3						
Low	181	64	1		1	
High	181	65	1.018 (0.718–1.443)	0.919	0.863 (0.595–1.422)	0.438
ABCC4						
Low	181	57	1		1	
High	181	72	1.489 (1.046–2.121)	0.026	1.479 (1.021–2.142)	0.038
ABCC5						
Low	181	50	1		1	
High	181	79	1.759 (0.234–2.508)	0.002	1.928 (1.318–2.820)	0.001
ABCC6						
Low	181	81	1		1	
High	181	48	0.495 (0.346–0.708)	<0.001	0.534 (0.366–0.778)	0.001
ABCC7						
Low	181	63	1		1	
High	181	66	1.185 (0.836–1.680)	0.340	1.077 (0.743–1.562)	0.695
ABCC8						
Low	181	57	1		1	
High	181	72	1.306 (0.920–1.853)	0.134	1.227 (0.843–1.785)	0.286
ABCC9						
Low	181	71	1		1	
High	181	58	0.757 (0.535–1.072)	0.116	0.794 (0.549–1.149)	0.221
ABCC10						
Low	181	60	1		1	
High	181	69	1.283 (0.907–1.815)	0.157	1.275 (0.883–1.841)	0.195
ABCC11						
Low	181	67	1		1	
High	181	62	0.893 (0.632–1.263)	0.523	0.832 (0.575–1.205)	0.331
ABCC12						
Low	181	58	1		1	
High	181	71	1.308 (0.923–1.852)	0.130	1.287 (0.891–1.859)	0.179
ABCC13						
Low	181	60	1		1	
High	181	69	1.313 (0.928–1.857)	0.112	1.261 (0.871–1.823)	0.220

Notes: § Adjusted for tumor stage. HR, hazard ratio; ABCC, ATP binding cassette subfamily C.

**FIGURE 4:**
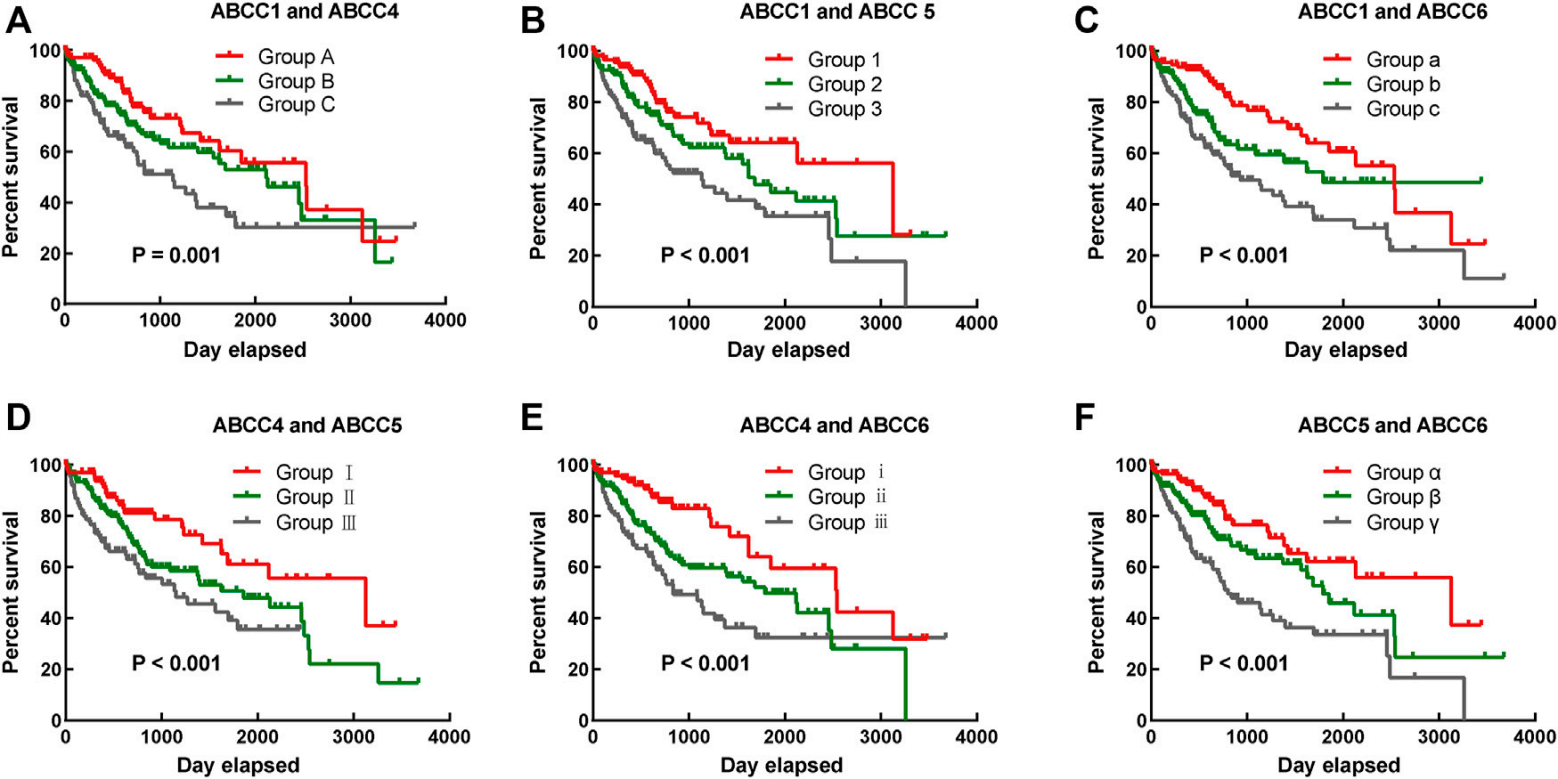
Combined effect survival analysis of *ABCC1*, *ABCC4*, *ABCC5*, and *ABCC6* in the TCGA database: **(A)** combined effect survival analysis of *ABCC1* and *ABCC4*, **(B)** combined effect survival analysis of *ABCC1* and *ABCC5*, **(C)** combined effect survival analysis of *ABCC1* and *ABCC6*, **(D)** combined effect survival analysis of *ABCC4* and *ABCC5*, **(E)** combined effect survival analysis of *ABCC4* and *ABCC6*, and **(F)** combined effect survival analysis of *ABCC5* and *ABCC6*. The details about the groups are displayed in Table 2.

To more accurately predict the prognosis of HCC patients, multivariate survival analysis was integrated into the combined effect survival analysis. In the combined effect survival analysis, it was observed that there was an even bigger prognosis difference among groups in combined effect survival analysis (Table 2). Group C with high expression of *ABCC1* and *ABCC4* was significantly correlated to bad outcome (*p* = 0.001, Figure 4A), so were group 3 with high expression of *ABCC1* and *ABCC5* (*p* < 0.001, Figure 4B), group c with high expression of *ABCC1* and low expression of *ABCC4* (*p* < 0.001, Figure 4C), group Ⅲ with high expression of *ABCC4* and *ABCC5* (*p* = 0.001, Figure 4D), group ⅲ with high expression of *ABCC4* and low expression of *ABCC6* (*p* < 0.001, Figure 4E), and group γ with high expression of *ABCC5* and low expression of *ABCC6* (*p* < 0.001, Figure 4F).

**TABLE 2:** Combined effect survival analysis of ABCC1, ABCC4, ABCC5, and ABCC6.

Group	ABCC1	ABCC4	ABCC5	ABCC6	Patients	No. of events	MST (days)	Crude HR (95%CI)	Crude P	Adjusted HR (95%CI)	Adjusted P δ
A	low	low			106	27	2,532	1		1	
B	low	high			150	54	2,116	1.474 (0.928–2.342)		1.500 (0.925–2.432)	
	high	low									
C	high	high			106	48	1,135	2.322 (1.445–3.731)	0.001	2.191 (1.328–3.614)	0.002
1	low		low		117	27	3,125	1		1	
2	low		high		128	47	1,685	1.564 (0.973–2.513)		1.637 (0.985–2.719)	
	high		low								
3	high		high		117	55	1,135	2.487 (1.568–3.945)	<0.001	2.572 (1.565–4.227)	<0.001
a	low			high	116	29	2,532	1		1	
b	low			low	130	41	1791	1.698 (1.053–2.739)		1.813 (1.098–2.993)	
	high			high							
c	high			low	116	59	931	2.574 (1.649–4.018)	<0.001	2.315 (1.447–3.704)	<0.001
Ⅰ		low	low		100	23	3,125	1		1	
Ⅱ		low	high		162	61	1852	1.767 (1.093–2.856)		1.897 (1.138–3.162)	
		high	low								
Ⅲ		high	high		100	45	1,149	2.528 (1.522–4.201)	0.001	2.790 (1.618–4.813)	<0.001
ⅰ		low		high	97	21	2,542	1		1	
ⅱ		low		low	168	63	1791	2.009 (1.221–3.307)		2.322 (1.379–3.910)	
		high		high							
ⅲ		high		low	97	45	837	2.988 (1.771–5.042)	<0.001	2.792 (1.595–4.887)	<0.001
α			low	high	111	25	3,125	1		1	
β			low	low	140	48	1791	1.628 (1.004–2.641)		1.641 (0.983–2.739)	
			high	high							
γ			high	low	111	56	802	2.850 (1.777–4.571)	<0.001	2.939 (1.772–4.874)	<0.001

Notes: δ Adjusted for tumor stage. MST, median survival time; No. of events, number of events; HR, hazard ratio; ABCC, ATP binding cassette subfamily C.

### Nomogram Based on *ABCC1*, *4*, *5*, and *6* and Tumor Stage

**FIGURE 5:**
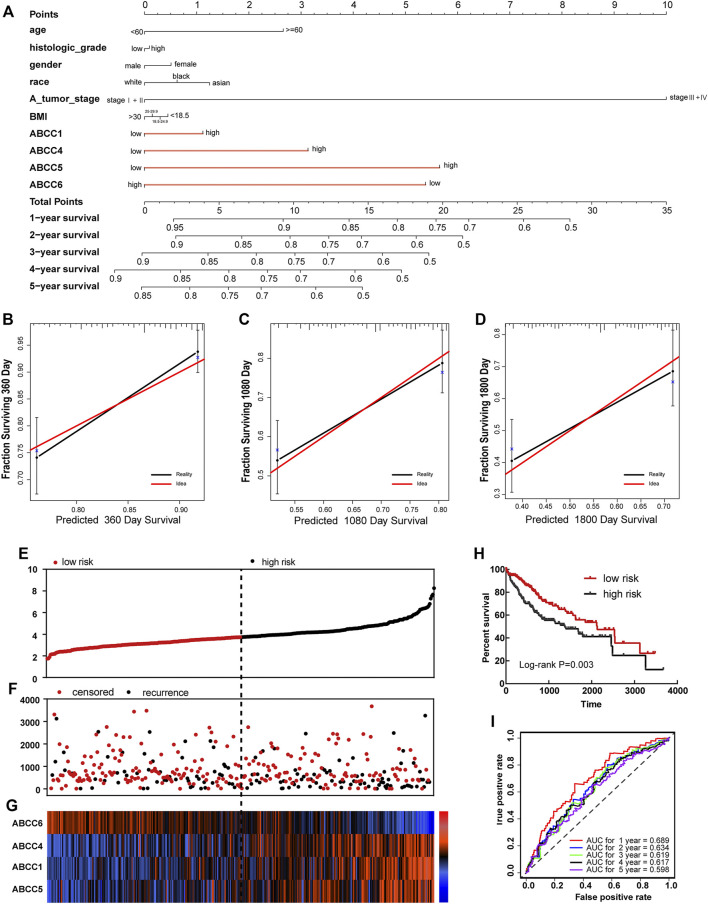
Nomogram and prognostic signature constructed in terms of *ABCC1*, *ABCC4*, *ABCC5*, and *ABCC6* in the TCGA database: **(A)** Nomogram based on expression of *ABCC* genes and clinicopathologic features; **(B)** internal validation for 1-year survival; **(C)** internal validation for 3-year survival; **(D)** internal validation for 5-year survival; **(E)**, scatter plot for risk score; **(F)** scatter plot for survival time (days); **(G)**, heat map corresponding to the expression of *ABCC1*, *ABCC4*, *ABCC5*, and *ABCC6*; **(H)** survival analysis for high- and low-risk score groups; and **(I)** AUC for inspecting the efficiency of the prognostic signature for predicting long-term prognosis.

In the survival analysis, we found that ABCC1.4.5.6 was strongly associated with the prognosis of HCC. In addition, the clinical factor tumor stage could also partially distinguish patients with good and bad prognoses. Thus, a nomogram integrating clinical elements and ABCC gene expression was constructed in terms of the COX proportional hazards model. In the nomogram, the contribution of *ABCC1*, *ABCC4*, *ABCC5*, *ABCC6*, and clinicopathologic features to the overall survival of HCC patients was displayed by virtue of the length of the scales (Figure 5A). The calibration plot for 1-, 3-, and 5-year survival after surgery revealed a satisfactory overlap between calculation and reality (Figures 5B–D).

### Prognostic Signature Based on the TCGA Database

In terms of the expressions of *ABCC1*, *ABCC4*, *ABCC5*, and *ABCC6*, the prognostic signature for HCC patients was built in the TCGA database and Guangxi cohort. Each HCC patient was assigned with a risk score in terms of the expression of *ABCC1*, *ABCC4*, *ABCC5*, and *ABCC6*. In the prognostic signature built for the TCGA database, the risk score for each patient was displayed in the upper scatter plot, and the patients were divided into two groups based on the median value (Figure 5E). The survival time and survival status of specific patients can be observed from the middle scatter plot, which showed that the dots representing patients in the high-risk group tended to cluster lower (Figure 5F). The expression levels of *ABCC1*, *ABCC4*, *ABCC5*, and *ABCC6* in patients were presented in the form of heat maps (Figure 5G). A significant difference in overall survival was observed between the high-risk and low-risk groups (Figure 5H, *p* = 0.003). The AUC value of the prognostic signature for 1-year, 3-year, and 5-year overall survival prediction was 0.689, 0.619, and 0.598, respectively (Figure 5I).

### Validation in the Guanxi HCC Cohort

**FIGURE 6:**
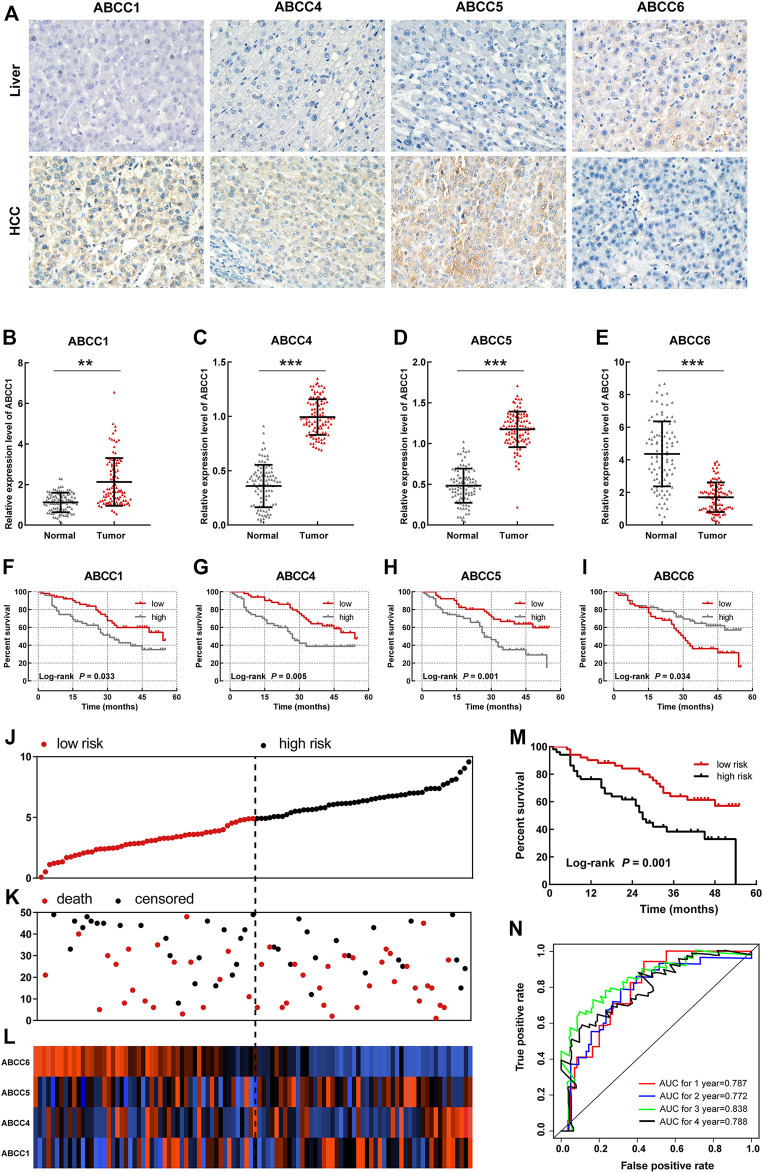
Validation for the prognostic significance of *ABCC1*, *ABCC4*, *ABCC5*, and *ABCC6* in the Guangxi HCC cohort: **(A)** expression of ABCC1, ABCC4, ABCC5, and ABCC6 in HCC tissues and paraneoplastic tissues assessed by IHC assays; **(B–E)** histogram showing ABCC1, ABCC4, ABCC5, and ABCC6 expression levels in HCC tissues and paraneoplastic tissues assessed by PCR assays; **(F–I)** survival curve of ABCC1, ABCC4, ABCC5, and ABCC6 in the Guangxi HCC cohort; the patients were grouped based on median expression; **(J)** Scatter plot for risk score; **(K)** scatter plot for survival time (months); **(L)** heat map corresponding to the expression of *ABCC1*, *ABCC4*, *ABCC5*, and *ABCC6*; **(M)** survival analysis for high- and low-risk score groups; and **(N)** AUC for inspecting the efficiency of the prognostic signature for predicting long-term prognosis.

A total of 102patients who were hospitalized in the first affiliated hospital of Guangxi Medical University from September 2016 to December 2018 were taken into the group for validation. The baseline information for these patients is presented in Table 3. The expressions of ABCC in HCC tissues and in paraneoplastic tissues were detected by immunohistochemical (IHC) and polymerase chain reaction (PCR) assays, respectively. In the IHC assay, the expressions of ABCC1, ABCC4, and ABCC5 in HCC tissues were significantly higher than that of paraneoplastic tissues, while ABCC6 was higher expressed in paraneoplastic tissues (Figure 6A). The same expression trends of ABCC1, ABCC4, ABCC5, and ABCC6 were observed at the mRNA level (Figures 6B–E). The prognostic significance of ABCC1, ABCC4, ABCC5, and ABCC6 was also observed in the Guangxi HCC cohort (Figures 6F–I; Table 4).

**TABLE 3:** Clinical characteristics of patients in HCC from Guangxi China.

Variables	Patients	Overall survival			
	(*n* = 102)	No. of events	MST (days)	HR (95% CI)	P
Age					
<60	76	37	23.9		
60	26	12	NA	0.956 (0.498–1.834)	0.891
Gender					
Female	14	5	NA		
Male	88	44	35	0.625 (0.247–1.583)	0.314
BMI					
<24.9	81	38	45		
>24.9	21	11	40	1.054 (0.539–2.063)	0.876
Alcohol					
No	65	34	33		
Yes	37	15	NA	1.595 (0.862–2.950)	0.131
Cirrhosis					
No	9	3	NA		
Yes	93	46	40	1.175 (0.544–5.637)	0.337
Child					
No	100	48	45		
Yes	2	1	3	6.122 (0.796–47.096)	0.045
BCLC					
A	67	32	45	1	
B	28	19	NA	3.757 (1.267–4.144)	
C	7	5	30	6.677 (5.878–8.201)	0.032
Missing	2				
AFP					
<200	50	18	NA	1	
>200	51	31	30	2.038 (1.139–3.648)	0.030
Missing	1				
Radical resection					
No	30	16	33	1	
Yes	70	32	45	0.807 (0.442–1.473)	0.480
Missing	2				
Histological					
Low	5	1	48	1	
Middle	65	32	35	2.978 (0.407–21.813)	
High	22	11	33	3.255 (0.419–25.275)	0.448

Notes: HCC, hepatocellular carcinoma; MST, median survival time; OS, overall survival; HR, hazard ratio; CI, confidence interval.

**TABLE 4:** Survival analysis results of ABCC genes in the Guangxi cohort.

Gene expression	Patients (*n* = 102)	Overall survival					
		No. of event	MST (months)	Crude HR (95% CI)	Crude P	Adjusted HR (95% CI)	Adjusted P ζ
ABCC1							
Low	51	20	54				
High	51	29	31	1.835 (1.036–3.251)	0.033	1.81 (0.998–3.283)	0.034
ABCC4							
Low	51	22	54				
High	51	27	27	1.991 (1.124–3.557)	0.005	1.912 (1.063–3.437)	0.03
ABCC5							
Low	51	17	NA				
High	51	32	27	2.895 (1.594–5.258)	0.001	2.750 (1.509–5.010)	0.001
ABCC6							
Low	51	28	31				
High	51	21	NA	0.582 (0.329–1.029)	0.034	0.065 (0.303–1.038)	0.046

Notes: ζ Adjusted for child pugh stage, BCLC, stage and AFP; NA, not available; MST, median survival time; HR, hazard ratio.

In the prognostic signature built for the Guangxi cohort, patients were divided into two groups in terms of the risk score (Figure 6J). The same as above-mentioned, the dots representing patients in the high-risk group also tended to cluster lower (Figure 6K). The expression levels of *ABCC1*, *ABCC4*, *ABCC5*, and *ABCC6* in patients were presented in the form of heat maps (Figure 6L). The prognosis of the high-risk group was significantly worse than that of the low-expression group (Figure 6M, *p* = 0.001). The AUC value of the prognostic signature for 1-year, 2-year, 3-year, and 4-year overall survival prediction was 0.787, 0.772, 0.838, and 0.788, respectively (Figure 6N). In the nomogram, the contribution of *ABCC1*, *ABCC4*, *ABCC5*, *ABCC6*, and clinicopathologic features to overall survival was displayed by the length of the corresponding scales (Supplementary Figure S4A). The calibration plot for 1-, 2- and 3-year survival after the surgery revealed a satisfactory overlap between calculation and reality (Supplementary Figures S4B–D).

### Biological Functional Exploration of *ABCC*s

**FIGURE 7:**
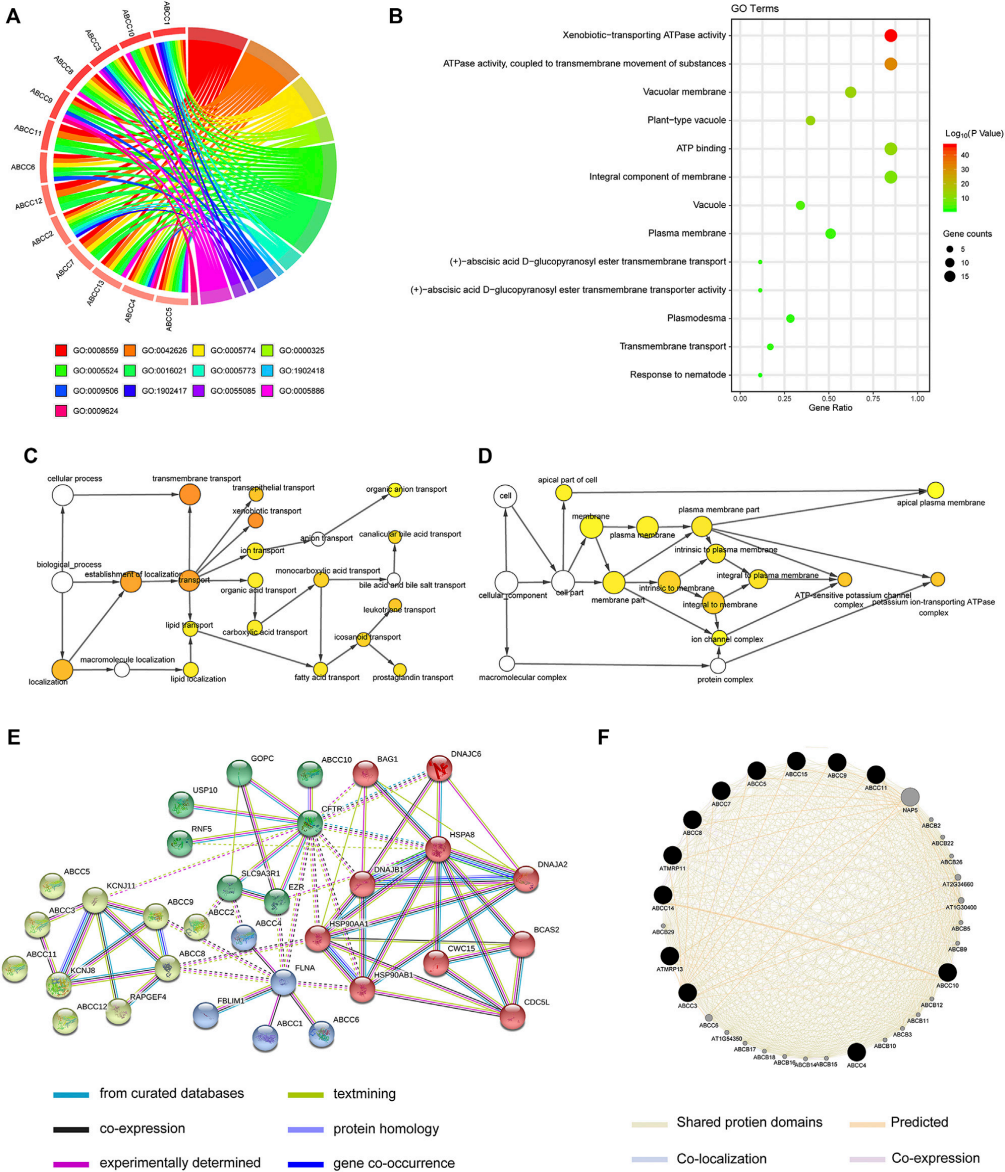
Biological function analysis for ABCC genes by bioinformatics: **(A)** circle plot for displaying the relationship between gene and GO term, **(B)** bubble plot for GO terms, **(C-D)** the network diagram of the relationship between enriched GO terms, **(E-F)** interactions of *ABCCs* that were analyzed from STRING and Genemania, respectively.

The enrichment analysis of the *ABCC* gene by setting Homo sapiens as the background was performed on the DAVID online database for obtaining enrichment information about GO terms. The corresponding relationship between ABCCs and GO terms is displayed in Figure 7A. The enrichment analysis of GO showed that *ABCCs* were mainly related to ATP binding, ATP activity, transmembrane, and other biological functions (Figure 7B). The bubble color from red to green represents the biological function of the –log (*p*-value) from high to low. The network diagram of the relationship between enriched GO terms is shown in Figures 7C,D. Interactions of *ABCCs* which were analyzed from STRING and Genemania are respectively displayed in Figures 7E,F.

### GSEA

**FIGURE 8:**
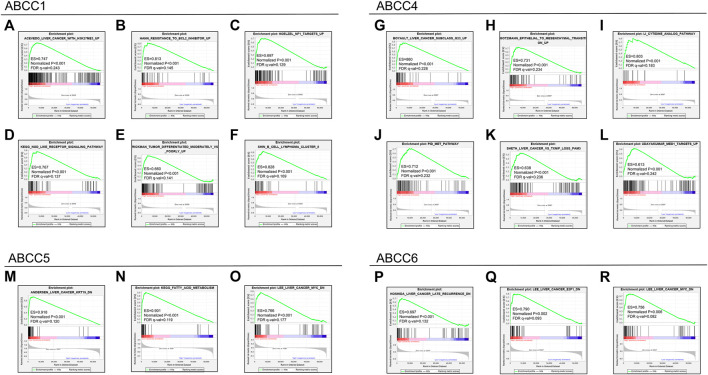
GSEA results for the *ABCC* genes in the TCGA database: **(A–F)** GSEA results for *ABCC1* in HCC, **(G–L)** GSEA results for *ABCC4* in HCC, **(M–O)** GSEA results for *ABCC5* in HCC, and **(P–R)** GSEA results for *ABCC6* in HCC.

The GSEA results revealed that the expression of *ABCC1* was associated with tumor differentiation, nod-like receptor signal pathway, resistance to the bcl2 inhibitor up, and so on (Figures 8A–F). The pathways that *ABCC4* might regulate are shown in Figures 8G–L. *ABCC5* might impact HCC by regulating the fatty acid metabolism and the expression of kt19 and myc (Figures 8M–O). The result of GSEA revealed that high expression of *ABCC6* was accompanied with lower HCC late recurrence (Figure 8P). It also illustrated that *ABCC6* might impact HCC by regulating E2F1 and myc (Figures 8Q,R).

### Correlation Analysis of ABCC Gene Expression and Tumor-Infiltrating Immune Cells

**FIGURE 9:**
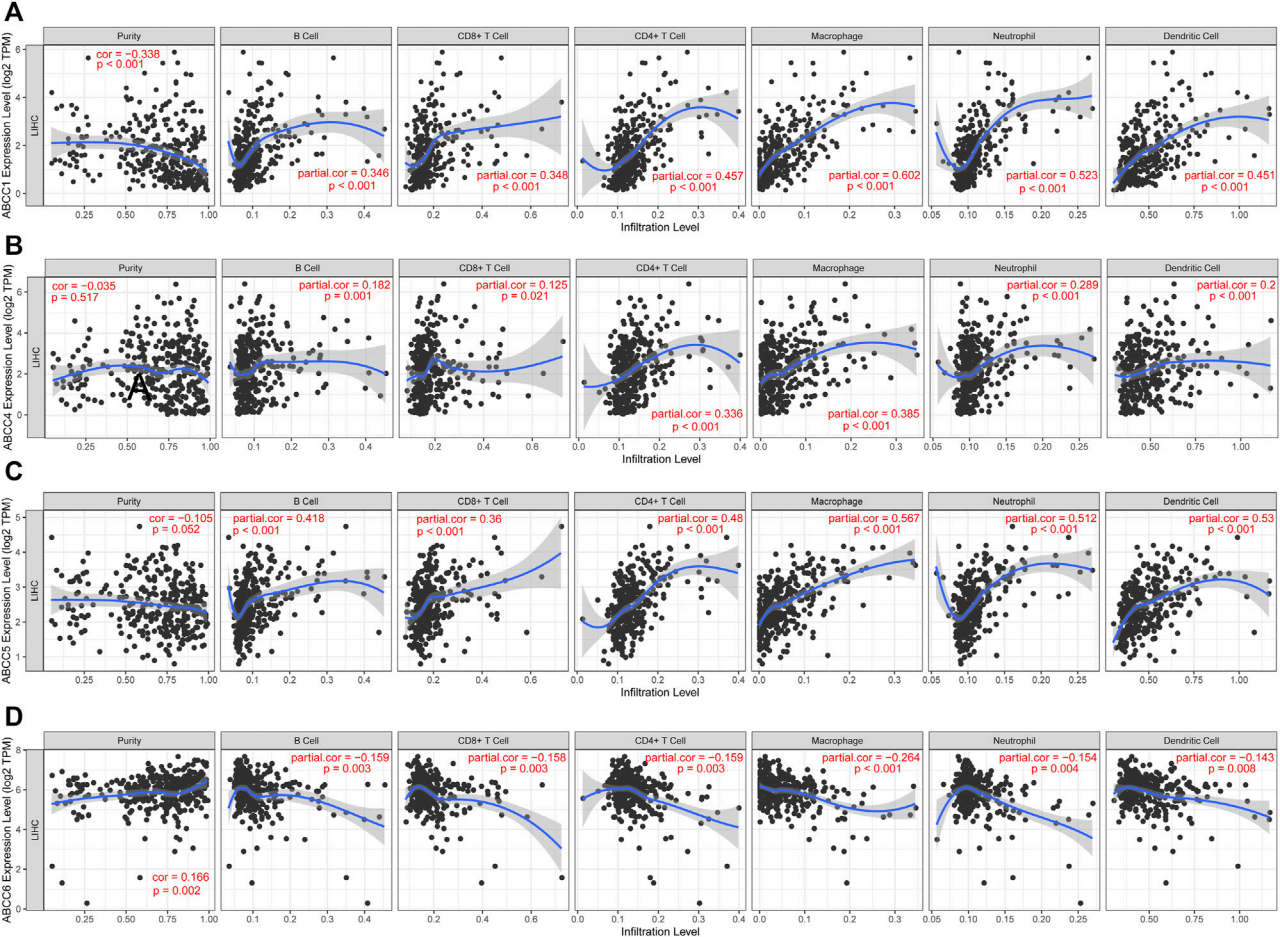
Correlation between ABCC gene expression and tumor-infiltrating immune cells: **(A)** scatter plot corresponding to ABCC1 expression and tumor-infiltrating immune cells, **(B)** scatter plot corresponding to ABCC4 expression and tumor-infiltrating immune cells, **(C)** scatter plot corresponding to ABCC5 expression and tumor-infiltrating immune cells, and **(D)** scatter plot corresponding to ABCC6 expression and tumor-infiltrating immune cells.

The estimation of the abundance of immune cell infiltration showed that *ABCC1*, *ABCC4*, and *ABCC5* were significantly positively associated with infiltration of immune cells, which include B cells, CD8^+^ T cells, CD4^+^ T cells, macrophages, neutrophils, and dendritic cells (Figures 9A–C). However, *ABCC6* was negatively associated with the infiltration of immune cells (Figure 9D).

## Discussion

*ABCC* expressions were analyzed in two data sets, and consistent results were obtained. Compared with normal tissues, they revealed that *ABCC1*, *ABCC4*, *ABCC5*, and *ABCC10* were significantly upregulated in HCC tissues, while *ABCC6* and *ABCC7* were significantly downregulated in HCC tissues. In the TCGA database, *ABCC5*, *ABCC7*, *ABCC9*, and *ABCC10* were equipped with high diagnostic efficacy for HCC (AUC > 0.8). In GSE76427, the good diagnostic efficacy for HCC was only discovered in *ABCC7*. Combining the results of the two data sets, we consider ABCC7 as a potential diagnostic marker for HCC.

In the TCGA database, *ABCC1*, *ABCC4*, *ABCC5*, and *ABCC6* were found to be associated with the prognosis of HCC, while further verification in GSE14250 indicated that only *ABCC6* was significantly correlated to the prognosis. The results of survival analysis in the two data sets were very similar, although not identical. We observed that the expression of *ABCC1* and *ABCC5* was associated with the prognosis of liver cancer in both data sets. The reason for the different conclusions may lie in the population difference and inconformity in the causes of neoplasm. HCC patients in GSE76427 were mainly in the Asian population, and the proportion of hepatitis B virus infection was high. However, the majority of HCC patients in the TCGA database were Caucasian and the proportion of hepatitis B virus infection was low. The sample size of both databases is relatively large, and the follow-up data were also of high quality. The results from both databases should be reliable but may apply to different populations. Both clinicopathologic features and biomarker expression were included in the nomogram as prognostic dependent variables, with the length of each variable clearly reflecting its contribution to the prognosis of liver cancer.

Based on the four prognostic biomarkers obtained from the survival analysis, we further performed combined effect survival analysis, nomogram, and prognostic signature based on biomarker expression. The combined survival analysis had obvious advantages, and the prognostic difference between groups was more remarkable. The length of each variable in the nomogram clearly reflects its contribution to the prognosis of liver cancer.

ABCC1 transports drugs to the extracellular substances, thereby reducing the drug concentration and generating drug resistance in cancer (Wlcek and Stieger, 2014). In the liver, ABCC1 undertakes excretion of the drugs into the bile (Zhou, 2008). The ontogeny, localization, expression, and function of ABCC1 in HCC were reported in several research studies, and the previous reports mainly focused on the role of ABCC1 in HCC drug resistance (Flens et al., 1996; Nies et al., 2001; Vander Borght et al., 2005). It was reported that ABCC1 was significantly upregulated in the tissues of oxaliplatin-resistant, 5-fluorouracil-resistant, and sorafenib-resistant HCC patients (Ding et al., 2017; Huang et al., 2018; Ding et al., 2019). In HCC, increased ABCC1 expression was related to increasing dedifferentiation, tumor size, and microvascular invasion (Vander Borght et al., 2008; Zhou, 2008).

Located on the inner surface of the basal side of the liver cells, ABCC4 undertakes bile salt transport (Borst et al., 2007). Previous studies have shown that ABCC4 expression is extremely low in the normal adult liver and fetal liver (Sharma et al., 2013), and *ABCC4* expression is significantly increased in cholestatic hepatocyte cell membranes (Gradhand et al., 2008; Sharma et al., 2013). Studies have shown that *ABCC4* is highly expressed in HCC tissues (Sekine et al., 2011; Borel et al., 2012; Luo et al., 2020). Recently, ABCC4 was found to play an important role in HCC oncogenesis and development promoted by decreasing the haploid of p53 (Luo et al., 2020). In addition, ABCC4 could specifically and independently distinguish the aggressive subtypes of HCC (Gradhand et al., 2008).

Here are a few reports on ABCC5 in HCC, with the existing relevant study indicating that ABCC5 is highly expressed in the liver cancer tissues. Our findings in this investigation also confirm this conclusion.

T lymphocytes are known as the main cells of the tumor immunity. Cytotoxic CD8+T cells play a particularly vital role in anticancer immune response (Vesely et al., 2011; Raskov et al., 2021). Once successfully activated, CD8+T cells secreted death-inducing granules to enhance the killing effect of target cells (Basu et al., 2016). Accumulating evidence indicates that TRM (tissue-resident CD8^+^ memory T cells) is essential for suppressing cancer growth. In a mouse model, whether generated during tumorigenesis or prior to tumor challenge, antitumor TRM cells revealed suppression in cancer growth (Park et al., 2019). Regulatory T cells inhibit anticancer immunity by preventing the protective immunosurveillance of neoplasia and hindering antitumor immune responses in tumor-bearing hosts, thereby promoting the tumor progression (Sakaguchi et al., 2010; Wing and Sakaguchi, 2010; Togashi and Nishikawa, 2017).

B cells have a crucial part in the regulation of T cell response against tumors (Olkhanud et al., 2011; Tadmor et al., 2011). There is a crosstalk between the B and T lymphocytes in antitumor immunity (Blair et al., 2010; DiLillo et al., 2010). Natural killer cells (NK cells) in cancer are involved in priming a multilayered immune response to achieving long-lasting immunity against tumors, in which T cells are involved (Morandi et al., 2012; Ferlazzo and Moretta, 2014). Moreover, NK cells generate cytokines and chemokines that regulate immune responses. The function of non-NK ILCs (innate lymphoid cells, ILCs) in cancer remains unclear.

Combining this investigation and previous research studies, we could preliminarily conclude that *ABCC1*, *ABCC4*, and *ABCC5* reduce drug sensitivity by influencing drug transport out of cells, thus resulting in a poor prognosis in these patients with HCC. In this study, we also found a significant positive correlation between *ABCC1*, *ABCC4*, and *ABCC5* expression and immune cell infiltration.

There were no reports on *ABCC6* in HCC before. The role of *ABCC6* in HCC is completely opposite to that of *ABCC1*, *ABCC4*, and *ABCC5*. We found that *ABCC6* expression was decreased in the liver cancer tissues, and the patients with low *ABCC6* expression had a better prognosis. We speculate that *ABCC6* may function through a completely different mechanism, and the specific findings need to be further studied.

## Data Availability

The raw data supporting the conclusion of this article will be made available by the authors, without undue reservation.

## Supplementary Material

Supplementary Figure 1 ABCC genes that were not found to be differentially expressed between HCC tissues and paraneoplastic tissues: **(A)** ABCC3, **(B)** ABCC8, **(C)** ABCC11, **(D)**, ABCC12, and **(E)** ABCC13.

Supplementary Figure 2 ROC curve for ABCC genes with average performance in diagnostic efficacy assessment: **(A)** ABCC1, **(B)** ABCC2, **(C)** ABCC3, **(D)** ABCC4, **(E)** ABCC6, **(F)** ABCC8, **(G)** ABCC11, **(H)** ABCC12, and **(I)** ABCC13.

Supplementary Figure 3 Survival curves of ABCC genes not found to be associated with HCC prognosis: **(A)** ABCC2, **(B)** ABCC3, **(C)** ABCC7, **(D)** ABCC8, **(E)** ABCC9, **(F)** ABCC10, **(G)** ABCC11, **(H)** ABCC12, and **(I)** ABCC13.

Supplementary Figure 4 Nomogram constructed based on *ABCC1*, *ABCC4*, *ABCC5*, and *ABCC6* in the Guangxi cohort. **(A)** Nomogram based on expression of *ABCC* genes and clinicopathologic features; **(B)** internal validation for 1-year survival; **(C)** internal validation for 2-year survival; **(D)** internal validation for 3-year survival.

Supplementary Figure 5 Workflow to summary manuscript.

## Abbreviations

ABCC, ATP binding cassette subfamily C; HCC, hepatocellular carcinoma; ROC, receiver operating characteristic curve; HBV, hepatitis B virus; HCV, hepatitis C virus; AUC, area under curve; GSEA, gene set enrichment analysis; IHC, Immunohistochemical.
