# Supplementary figures and images for: Clinical Significance and Potential Mechanisms of ATP Binding Cassette Subfamily C Genes in Hepatocellular Carcinoma
Source: Front Genet. 2022 Mar 7;13:805961. doi: 10.3389/fgene.2022.805961 (PMC8948437; doi:10.3389/fgene.2022.805961)

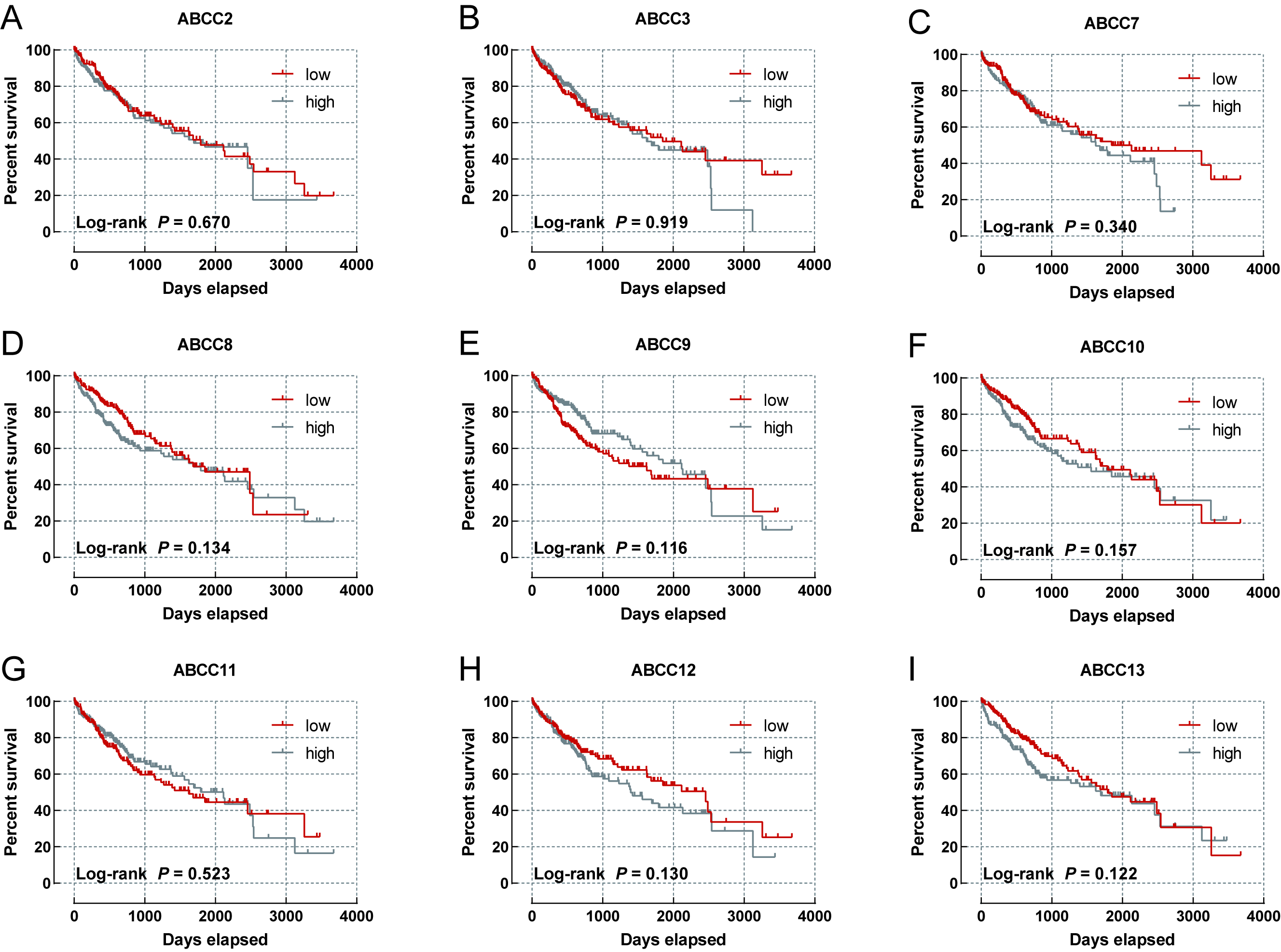

Supplement: Supplementary file 3 [file Image3.TIF]

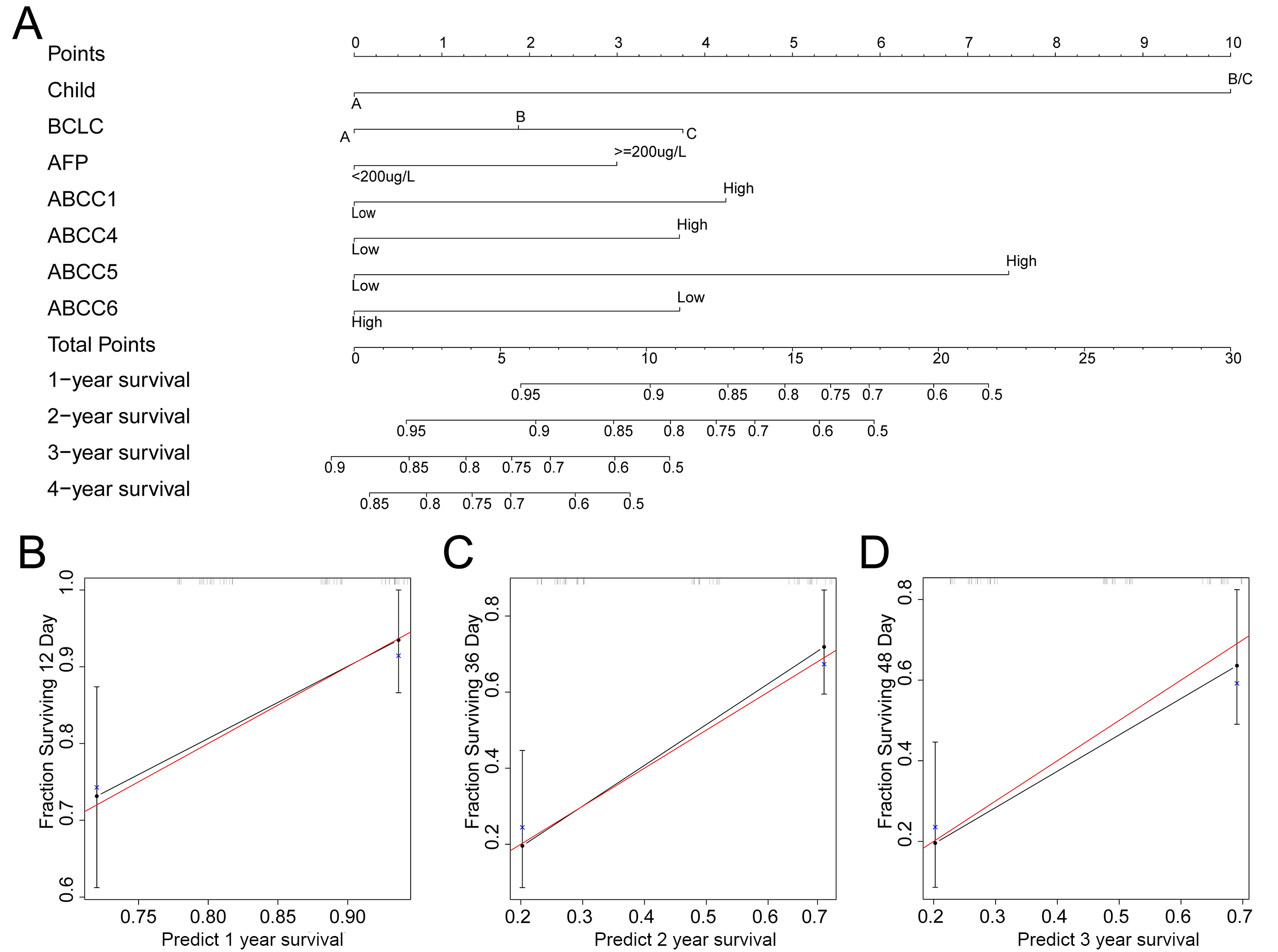

Supplement: Supplementary file 4 [file Image4.TIF]

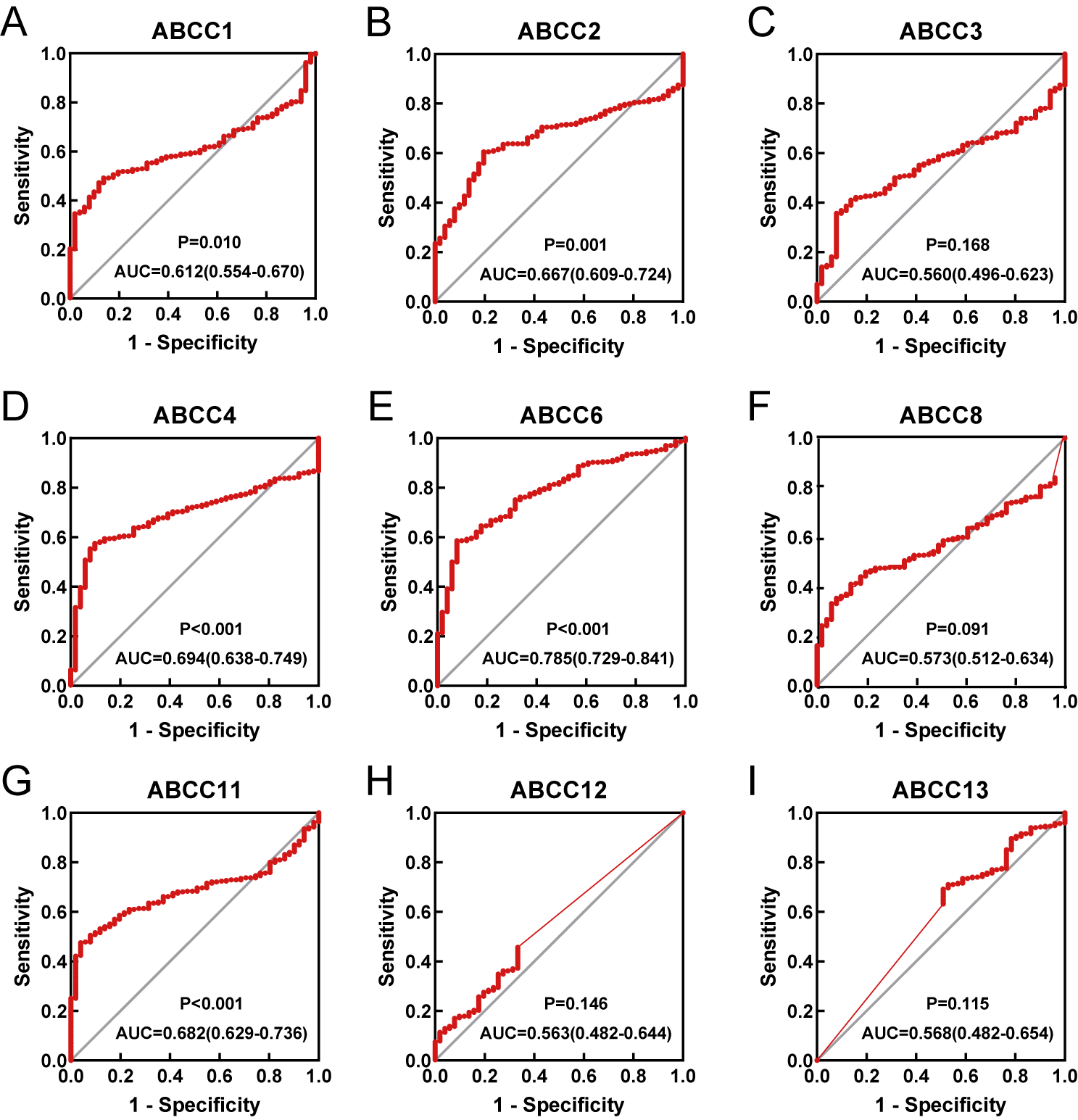

Supplement: Supplementary file 5 [file Image2.TIF]

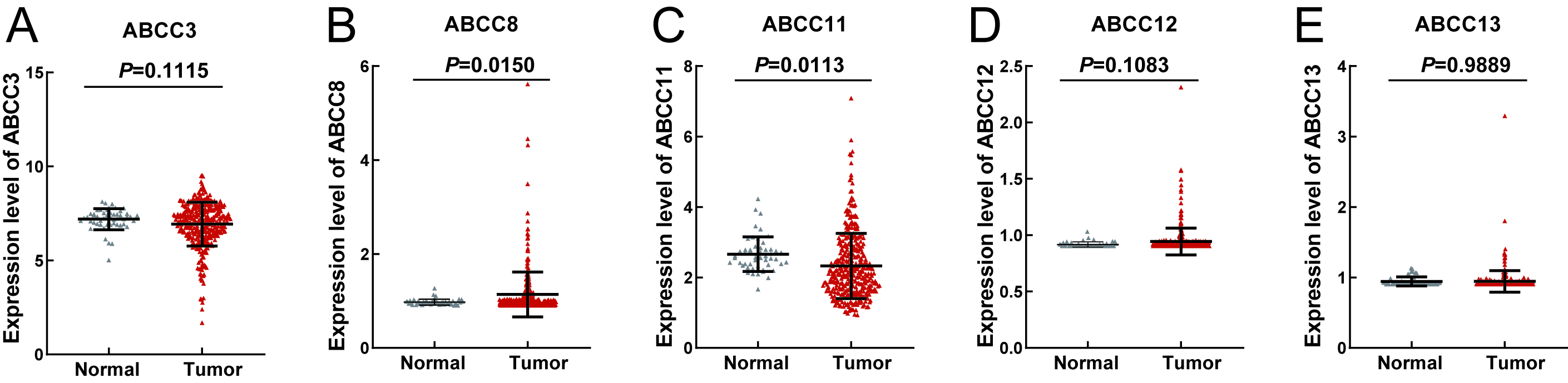

Supplement: Supplementary file 6 [file Image1.TIF]

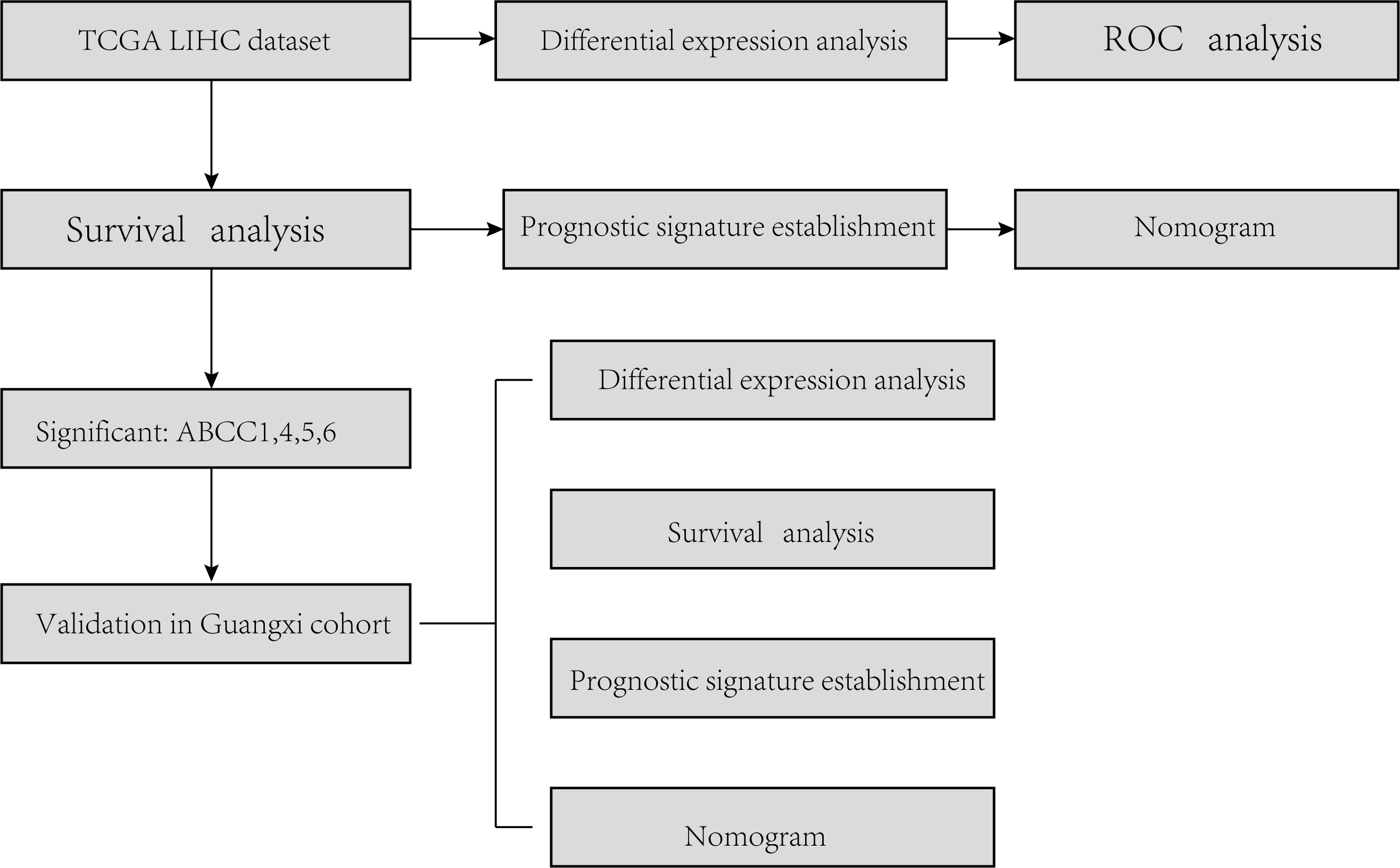

Supplement: Supplementary file 9 [file Image5.TIF]
